# Health literacy among migrants in Germany – results of the quantitative cross-sectional HLS-MIG study: Eva-Maria Berens

**DOI:** 10.1093/eurpub/ckac129.723

**Published:** 2022-10-25

**Authors:** E-M Berens, J Klinger, S Carol, D Schaeffer

**Affiliations:** 1 Institute for Sociology and Social Psychology, University of Cologne, Köln, Germany; 2 Interdisciplinary Centre for Health Literacy Research, University Bielefeld, Bielefeld, Germany; 3 School of Sociology, University College Dublin, Dublin, Ireland

## Abstract

**Background:**

Health literacy (HL) is considered an important prerequisite for health. HL research often identifies migrants as vulnerable for low HL. However, in-depth data on HL among migrants and its determinants are still scarce, especially in Germany. Therefore, the analysis presents first time data on HL among migrants in Germany, specifically in the domains of health care, disease prevention and health promotion, considering migrant-specific and universal factors, such as social and psychological aspects.

**Methods:**

Around 1.000 first- and second-generation adult migrants from two of the largest migrant groups in Germany, from Turkey and former Soviet Union (FSU), were interviewed face-to-face in German, Turkish or Russian in late summer 2020. HL was measured using the HLS19-Q47 instrument. Bi- and multivariate statistical analyses were conducted.

**Results:**

More than half of the migrants have limited general HL on average. HL in disease prevention and health promotion was lower than in health care. Low social status, financial deprivation, low literacy skills and low self-efficacy were negatively correlated with each HL domain. Social integration, by contrast, goes along with a higher HL. Duration of stay in Germany and country of origin were only partly associated with HL.

**Conclusions:**

Our study goes beyond existing studies by analyzing HL in its domains and including the explanatory power of self-efficacy and social integration for migrants’ HL. Moreover, we reveal that migrants can't generally be considered as vulnerable, as oftentimes outlined. There is need for targeted interventions, e.g. enhancing usability of health information, for the abovementioned subgroups regarding differences in domains.

**Key messages:**

• HL among migrants varies across domains and is only partly linked to migrant-specific features but mainly associated with universal aspects.

• Measures to increase HL should consider differences in domains and address vulnerable subgroups including targeted interventions, respectively.

